# Should they stay, or should they go? Relative future risk of bovine tuberculosis for interferon-gamma test-positive cattle left on farms

**DOI:** 10.1186/s13567-015-0242-8

**Published:** 2015-09-04

**Authors:** Angela Lahuerta-Marin, Martin Gallagher, Stewart McBride, Robin Skuce, Fraser Menzies, Jim McNair, Stanley W. J. McDowell, Andrew W. Byrne

**Affiliations:** Veterinary Science Division, Department of Bacteriology, Agri-food and Biosciences Institute, Stormont, Belfast, BT4 3SD UK; Veterinary Epidemiology Unit, Department of Agriculture and Rural Development, Dundonald House, Stormont, Belfast, UK; School of Biological Sciences, Queen’s University Belfast, Belfast, BT7 1NN UK

## Abstract

Bovine tuberculosis (bTB), caused by *Mycobacterium bovis*, is a serious infectious disease that remains an ongoing concern for cattle farming worldwide. Tuberculin skin-tests are often used to identify infected animals (reactors) during test-and-cull programs, however, due to relatively poor sensitivity, additional tests can be implemented in parallel. For example, in Northern Ireland interferon-gamma (IFN-g) testing is used in high-risk herds. However, skin-test negative animals which are positive to the IFN-g test are not required by law to be slaughtered – therefore the final choice for these animals’ fate is left with the owner. During this study we investigated whether these animals represented a greater risk of becoming a skin reactor, relative to IFN-g test negative animals from the same herds. Our study population included 1107 IFN-g positive animals from 239 herds. A Cox-proportional hazard model indicated that animals which tested IFN-g positive were 2.31 times (95% CI: 1.92-2.79; *P* < 0.001) more likely to become a reactor compared with IFN-g negative animals. Animals from dairy herds, and from herds in the south-east, were of higher risk than animals from beef herds and other regions, respectively. Our findings suggest that IFN-g positive animals represent a higher risk of failing a skin-test in the future, indicating the value of IFN-g testing for identifying early-stage infected animals. These IFN-g positive animals are not under any disease restriction, and may move freely (trade), which may put recipient herds at increased risk. Our findings provide important evidence for stakeholders engaged in bTB eradication programs.

**Electronic supplementary material:**

The online version of this article (doi:10.1186/s13567-015-0242-8) contains supplementary material, which is available to authorized users.

## Introduction

Bovine tuberculosis (bTB) is a chronic and complex disease caused by *Mycobacterium bovis* infection, with cattle being the primary host [1–4]. The disease still remains a significant animal health problem for cattle herds worldwide [5–9] with important trading and associated economic costs [10].

Reliable diagnostic methods are an essential part of an effective disease control or eradication scheme [3,11]. The ante-mortem diagnostic tests currently available to detect *Mycobacterium bovis* are imperfect [3]. The tuberculin skin-test is the statutory test performed within European member States as part of the eradication programmes under the 64/432/EEC directive [12]. This method has a good average specificity (99%-100%) but a relatively poor average sensitivity (51-80%) [3,13,14]. Thus, the method is generally prone to false negatives. This scenario is of particular concern when the tuberculin skin-test is applied to herds with a history of persistent bovine tuberculosis, as the test may fail to detect all infected animals, leaving an unknown burden of infection within the herd (residual infection) [15–17].

The gamma interferon (IFN-g) test was first described by Wood et al. in 1991 [18] and was approved by the European Commission in 2002 to be used as an ancillary test to the tuberculin skin-test with the main objective of maximising the detection of infected bTB animals (Regulation EC/1226/2002 amending Annex B to Directive 64/432/EEC). Both tests can be used in series or in parallel, depending whether specificity (in series) or sensitivity (in parallel) for detection is prioritised. The performance characteristics of the IFN-g are also imperfect but complementary to the tuberculin skin-test, as this test has a better sensitivity ranging 88-94% but poorer specificity with a range of 85-98% relative to visible lesions [3]. Therefore, the test might be prone to false positives.

In Northern Ireland, where cattle are an important component of the local economy, bTB is considered endemic with a herd incidence of 6.4% in 2013 [19]. It is estimated that the official eradication scheme for bTB in Northern Ireland has an approximate cost of £30 million per annum [5]. Since 2004, the IFN-g test has been used in Northern Ireland as an ancillary test in parallel with the tuberculin skin-test (single intradermal comparative tuberculin test, SICTT) in risky herds with the main aim of identifying more infected animals. In 2013, there were 215 herds with a total of 16 930 bovine animals tested with IFN-g in Northern Ireland [5]. The test is performed during bTB breakdowns that meet certain criteria defined by the Department of Agriculture and Rural Development (DARD). Specific criteria included non-pedigree herds of less than 200 cattle that had a current confirmed bTB herd breakdown were eligible for parallel herd testing with IFN-g and SICTT on a voluntary basis. More specifically, herds were included if: 1. they had a chronic history of bTB infection, having three or more herd SICTTs during which SICTT reactors were declared over the previous two years; 2. they had a SICTT where six or more SICTT reactors were disclosed; 3. they had an animal at routine slaughter (not a SICTT reactor animal) that had confirmed bTB (defined as either positive for *M. bovis* on bacteriological culture and/or following histopathological examination).

If positive animals to the IFN-g test are detected, no official disease restrictions apply to this group of animals. Thus, the herd owner has the last decision on the fate of such animals; whether they should be moved out of the herd to slaughter, be sold out, or to remain in the herd. It is believed that the NI scheme is unique in terms of IFN-g positive animal risk management compared to other bTB endemic countries (for example, in England IFN-g positive animals are under compulsory slaughter [20] and in Republic of Ireland (ROI) there is also a legal basis for compulsory removal of IFN-g positive animals (Statutory Instruments: No. 308/1989; 161/2000)).

At present, it is unknown whether IFN-g positive animals left within their herds in Northern Ireland represent a future risk for failing the skin-test, and consequentially causing a future breakdown for the herd owner. Previous research from the ROI has found increased risk for INF-g positive animals left on farm over short temporal periods (18 months) [21,22]. However, these previous studies utilised only moderate sample sizes (*n* = 26 herds), and used only univariable analyses. Here we have used a large dataset (*n* = 239 herds) of INF-g tested herds in Northern Ireland and utilise a multivariable survival analysis approach, to increase robustness. The main aims of the study were to assess the proportion of IFN-g positive animals left on farm and whether they represented a higher future risk for bTB compared with negative animals.

## Materials and methods

### Study population

Our study population comprised 239 herds with at least one IFN-g positive animal retained within it (i.e. an IFN-g positive animal that was not slaughtered up to 2 months after the test date) between 2004 and 2010 (animal were followed-up to 2014 before censoring thereafter if still alive). From such herds, two cohorts of animals were eligible for inclusion in the study: those with an IFN-g positive result and those with an IFN-g negative result from the same herd test and that remained on the farm for at least two months after the IFN-g test. Each animal was observed from the date of the IFN-g test until the date of a subsequent positive skin-test or the date of their last skin-test within the study period (five year follow-up), whichever occurred first. Some exclusion criteria were applied to identify “study animals”. Any animal that went to market (had a market move), or to another farm prior to a skin-test, was censored at that point. Therefore, any test after the first move of an animal was not of interest. Moreover, animals that were introduced into the herd after the initial IFN-g test were not included in the analysis.

### Data source and management

The final dataset was made available from the Department of Agriculture and Rural Development database system (APHIS) using Microsoft Access® and Cognos®. Data were extracted at an animal level from various herd and animal tables within APHIS using Access and aggregated into a single record per subject using Stata version 11 (Statacorp LP, College, Texas, USA). The data were analysed using Stata.

### Data analysis

#### Overview

A survival model was built to compare the risk of bTB outcome at skin-test (i.e. of failing the skin-test) between animals that had a positive IFN-g test relative to those that had a negative IFN-g test, while controlling for other risk factors. The outcome of interest was time to subsequent positive skin-test. A Cox proportional-hazard shared frailty model with a random effect for herd (clustering at the herd level) was used; the shared frailty was used to control for correlation amongst cows within herds. Independent variables of interest were Herd size (mean herd size), Herd type (dairy and beef), Sex (male and female) and Location. Location was derived from the Divisional Veterinary Offices (DVO) regions of Northern Ireland, which were grouped into 3 categories: North (Derry, Larne, Ballymena and Coleraine), Southwest (Omagh, Enniskillen and Dungannon) and Southeast (Newtownards, Newry and Armagh).

#### Univariable analysis

Chi-square tests were performed to assess associations between categorical independent variables and the proportion of animals in each cohort that became skin-test positive before the end of the study period. Kaplan-Meier survival curves were created for our outcome by both groups and for each categorical risk factor. Log-rank tests and Wilcoxon tests were used to compare survival times across the two groups of animals [23] and to explore whether or not to include each predictor in the final model. A univariable screening approach was used to select variables for consideration in a Cox proportional-hazards model using STCOX in Stata version 11. Variables with a *P*-value of <0.2 were considered for inclusion in the full multivariable model.

#### Multivariable analysis

The Cox proportional hazards model with shared frailty was fitted to adjust for clustering at herd level. A backward selection procedure was used to eliminate terms from the full model based on a likelihood ratio test (*P* > 0.05). Models were compared using the AIC (Akaike Information Criteria); amongst competing models, the model with the smallest AIC was considered the preferred model. The proportional hazards assumption was tested using a plot of –log (-log) survival lines, to examine if they were parallel and by examining the Schoenfeld residuals [24]. Kaplan-Meier curves were also used to test proportionality of predictors. Cox-Snell residuals and deviance residuals were computed to assess overall model goodness-of-fit. We repeated the multivariable model restricting our data to a follow-up period of 18 months (versus five year follow-up), in order to compare our findings with a previous study from the Republic of Ireland [21,22].

## Results

There were 4606 animals positive to the IFN-g test. However, of those a total of 1146 positive animals were left on farm (25%). In addition, 39 (3.4%) out of those 1146 animals were sold or moved out of the farm before the next tuberculin test after the disclosure of IFN-g results. Thus, the final cohort of IFN-g test-positive and SICTT negative animals left on farm was 1107, from the study population of 239 herds. These 239 herds contained a total of 22 820 animals. The remaining 21 713 animals were negative to the IFN-g tests.

Overall, the median number of IFN-g positive animals left on farm was 8 (Mean: 11.54; SD: 9.40). The median number of IFN-g positive animals left on farm from the northern region was 8 (mean: 13.16; SD: 10.56), in the south-western the median was 7 (Mean: 10.09; SD: 9.55), while in the south-eastern region the median value was 10 (mean: 11.66; SD: 8.26).

Of the 1107 IFN-g test positive animals left on the farm, the majority were female (91% of total) and from dairy herds (68% of total). There were also more of these animals from the south-east region (41%), in comparison to the south-west (33%) and the northern region (26%) (Table 1). A breakdown of the numbers of animals by gender and herd-type for the IFN-g negative dataset is presented in Additional file 1.

**Table 1:** **Breakdown of positive IFN-g animals according to a herd-type, gender and region location**

Region	Dairy		Beef		Total
	Male (%)	Female (%)	Male (%)	Female (%)	(%)
N	3 (1)	227 (78)	13 (4)	48 (17)	291 (100)
SW	3 (0.8)	216 (59)	34 (9)	114 (31)	367 (100)
SE	7 (2)	297 (66)	44 (10)	101 (22)	449 (100)
Total	13 (1)	740 (67)	91 (8)	263 (24)	1107 (100)

**Figure 1:**
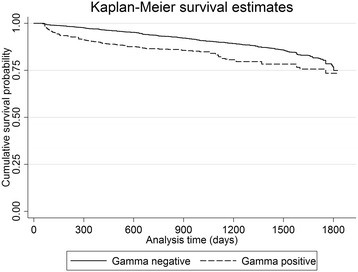
**Kaplan Meier survival estimates of time to subsequent positive skin-test, by Exposure (Gamma negative, Gamma positive).** There was a significant increased future risk of animals failing a future skin-test for bovine tuberculosis if they tested positive to an interferon gamma test during 2004-2010 using data from a cohort of cattle from Northern Ireland.

Overall, 14.3% and 6.6% of animals which were IFN-g positive and negative, respectively, had a subsequent positive skin-test during a five year follow up period (χ^2^ = 84.5, *P* < 0.001, Table 2). The Kaplan-Meier probabilities of surviving up to 5 years without failing the skin-test for IFN-g positive, and IFN-g negative animals, respectively, were as follows; Year 1: 0.90 and 0.97, Year 2: 0.86 and 0.94, Year 3: 0.84 and 0.90, Year 4: 0.78 and 0.86, and Year 5: 0.73 and 0.75 (χ^2^ = 54.09, *P* < 0.001). The Kaplan-Meier survival curves, for IFN-g positive and negative animals, are presented in Figure 1. Based on univariable model results, three of the independent variables were eligible (*p* < 0.2) for inclusion in the multivariable model. These were the three variables considered during multivariable model building: Exposure variable (Gamma positive or negative), Herd type, and DVO region.

**Table 2:** **The univariable association between the percentage of IFN-g tested animals that had a subsequent positive skin-test and independent variables**

Variable	Class	*N*	*N* with subsequent positive skin-test	% Skin-test positive	*p*-value (χ^2^ test)
Gamma	Negative	21713	1323	6.55	<0.001
	Positive	1107	138	14.26	
Herd	Beef	8342	400	5.15	<0.001
	Dairy	14478	1061	7.97	
DVO	North	6478	281	4.62	<0.001
	Southwest	7113	427	6.61	
	Southeast	9229	753	8.78	
Sex	Male	3409	75	2.21	<0.001
	Female	19411	1386	7.87

After model building, the final Cox-proportional hazards model contained three variables including the exposure, outcome of IFN-g test (positive/negative) (*P* < 0.001), herd type (*P* = 0.011), and DVO region (*P* = 0.019) (Table 3). There was a significant difference (*P* < 0.001) in survival times between animals that had a positive IFN-g test and animals that had a negative IFN-g test conditional on the frailty (i.e. from within the same herd). Animals which tested IFN-g positive and remained on the farm were 2.31 times (95% confidence interval: 1.92-2.79) more likely to have a positive skin-test during the subsequent study period compared with animals that were IFN-g negative from the same herd (Table 3). The hazard varied across locations with the south-east DVO region having a significantly higher risk than the southwest and north DVO regions, and an increased, although non-significant, risk in the southwest relative to the north (Table 3). The hazard of a future positive skin-test was significantly greater in dairy herds relative to beef herds (Table 3). There was strong statistical evidence (LRT χ^2^ = 1721, *P* < 0.001) that the frailty component contributes to the model and that there were within herd correlations among cows. The variance component was modelled on a log normal scale. None of the covariates varied over time. The graphical evaluation of proportionality implied that the assumption of proportionality was met. There was good agreement between the plotted hazard function and the expected 45° line of Cox-Snell residuals, indicating a good model-fit.

**Table 3:** **Final Cox-proportional hazards model of time to a subsequent positive skin-test**

Covariates	Hazard ratio	*P*-value	95 % Confidence interval	
Exposure (referent: Gamma negative)			Lower	Upper
Gamma positive	2.31	<0.001	1.92	2.79
Herd type (referent: Dairy)				
Beef	0.78	0.023	0.63	0.97
DVO region nreferent: Southeast)				
North	0.45	0.009	0.25	0.82
Southwest	0.56	0.030	0.33	0.95
Variance component				
Theta	2.66	<0.001		

The multivariable survival model with a restricted follow-up period (18 months/550 days) is presented in Additional file 2. Animals which tested IFN-g positive and remained on the farm were 3.69 times (95% confidence interval: 2.90-4.68) more likely to have a positive skin-test during the subsequent restricted follow-up period (18 months) compared with animals that were IFN-g negative from the same herd. This corresponded to 22.6% and 6.1% of animals which were IFN-g positive and negative, respectively, that had a subsequent positive skin-test during an 18 month follow-up period (χ^2^ = 187.63, *P* < 0.001). Beef herds were at significantly lower risk in comparison with dairy herds; however there was weak evidence of significant variation across regions using this restricted dataset.

## Discussion

The IFN-g test has proven to be a good ancillary test that has worked well in ongoing eradication programmes [25,26], albeit at the risk of disclosing false positives [27]. It is estimated that this test can detect infected cases earlier than the SICTT [28]. Both tests tend to target different populations of animals, thus they can complement each other to enhance the number of positive animals detected (Lahuerta et al., unpublished data; [3]), though at the risk of culling uninfected animals due to lower specificity relative to the skin test. As part of eradication schemes in many countries, animals that are IFN-g test positive are also culled [20,26]. Northern Ireland has a unique scheme for IFN-g testing because, 1. the cut-off of the test in use is lower than the recommended manufacturer (0.05 vs 0.1) to maximise sensitivity, 2. There is no official disease-restrictions on those IFN-g positive animals thus the culling of IFN-g positive animals is not compulsory. Consequently, the farmer has the last word on the animal’s fate – a choice likely influenced by the farmer’s perception of risk, and potentially the value of the IFN-g positive animal. Prior to this study, the future risk (if any) for bTB disclosure for these animals was not well understood, at the animal or herd level. This study presented a unique opportunity to follow a large sample of these positive animals left on farm and evaluate their risk of failing the SICTT compared with in-herd negative fellow animals (as a relative proxy for bTB risk).

This study has shown that IFN-g positive animals, left on farm, had a 2.3 times higher risk of failing the skin-test during the five year follow-up period compared with negative animals from the same herds with chronic bTB problems. There is sufficient evidence to suggest that this IFN-g positive group present a higher risk for a bTB breakdown in the future. This finding is of importance because this group of animals may be a source of infection to the home herd, neighbouring herds (through contiguous spread), local wildlife and trading herds associated with the home herd. Our findings are congruent with a previous smaller scale study from the Republic of Ireland [21,22]. During that study, INF-g positive animals (*n* = 26 herds) were monitored using the SCITT for up to 18 months after the initial test [21,22]. The authors reported that SICTT negative–IFN-g positive animals had 7–9 times greater odds (odds ratio from a 2x2 table) of becoming SICTT positive at follow-up relative to SICTT negative–IFN-g negative animals [21]. This equated to 28.6% of IFN-g positive animals failing a SCITT, relative to 4.4% of IFN-g negative animals, at follow-up [22]. Similar results were found during our study when the follow-up period was restricted to 18 months; 22.6% for IFN-g positive animals versus 6.1% for IFN-g negative animals. The overall effect of the IFN-g test status waned over time during our study – probably related to other factors influencing the probability of animals failing SCITT.

At the herd level, the future disclosure of a skin-test positive animal would trigger a herd breakdown, which incurs restrictions to trade for the affected farmer. This is of a particular concern as our study population comprised herds with a history of bTB, where it is possible that not all infected animals were cleared during breakdown periods. During our study period 39 IFN-g positive animals moved away from the farm of origin shortly after testing as they were not under any disease specific restriction. Previous work has found that animal movements were associated with increased risk for bTB [29–34]. The movement of positive IFN-g animals to clear herds could be particularly risky for disease introduction. Farmers currently cannot mitigate this risk, as information on an animal’s IFN-g test history is not readily available at market or to a potential private buyer. These findings also question the cost-benefit efficiency of these types of schemes in the bTB eradication program, where positive animals to the IFN-g test are not under any official disease restriction. More in-depth research on this is necessary to evaluate the current scheme as applied in Northern Ireland.

Location has been described previously as a risk factor for bTB in other countries such as the Republic of Ireland and England [29,35], with evidence of considerable spatial heterogeneity for bTB risk [36,37]. The south-east area in Northern Ireland (including the DVOs: Newry, Armagh and Newtownards) is one of the areas of the region with the highest levels of positive herds and positives animals within herds (Lahuerta-Marin et al. unpublished data). Herds from this region tend to be of small size and of high turnover in many cases, associated with intensive trading activities [5]. Recently, this region has been a “hot-spot” with the highest incidence of bTB in Northern Ireland [19]. We found that IFN-g positive animals within herds in this region were also at a higher risk of future SICTT failure, relative to other regions.

We found that IFN-g positive dairy animals had a higher risk of failing the tuberculin skin-test compared to animals from beef herds, and a similar result has been found elsewhere [25]. Due to the specific management practices, and also because bTB is a chronic disease, the productive life of dairy cows can be longer than beef cows. This may allow for greater exposure to the pathogen over time (i.e. dairy animals can accumulate greater time at risk; [4,32]). In addition, the longer residence of dairy cows within the herd may increase the potential for animal-to-animal transmission of any infected animals in comparison with beef herds. Furthermore, the life histories of dairy herds may influence immunological responses differentially, relative to other herd types [38].

A shared frailty model was selected to analyse these data. This is an extension of survival models whereby the random component, or frailty, is used to account for heterogeneity among groups of individuals or within an individual [39]. Frailties are shared across groups (clusters) of observations, thus allowing those observations within the same group to be correlated [40]. The frailties across different clusters account for unexplained variability at the cluster (herd) level. The key idea of these models is that individuals have different frailties, and the most frail will die (fail) earlier than the less frail [41]. There is sufficient evidence of a frailty effect in our data and that a shared frailty model fits the data well. The variance component theta informs us of the relativity of the outcome that corresponds to the random effect. The high significance of theta indicates strong heterogeneity between herds in terms of risk. Previous work has also found that the performance of the IFN-g test can vary across herds, with significant clustering within farms [27]. Some farms are highly risk averse relative to other farms and this could have important implications in the future in order to target higher risk herds.

The specificity of the IFN-g test is moderate (86%-99%) and can be prone to disclosing false bTB positive animals with an associated high economic cost [3,42]. Therefore, there may be a proportion of false positives in our cohort. This uncertainty with regards to infection status could have influenced the farmer’s decision not to send the animal to slaughter. Despite this specificity issue, our results showed that IFN-g positive animals can be of increased risk for bTB disclosure, relative to IFN-g negative animals and therefore should be considered a risk.

A limitation of this study was that most of the positive animals left in the herd were females mainly because of the predominant farming types (dairy and breeding beef farms) in the study. There might be some ascertainment bias also due to the voluntary basis for participating in the scheme. Hence, the results may be more applicative to chronic herds and may not be representative of the whole cattle farming industry in Northern Ireland. However, we consider the study to be sufficiently robust to inform farmers, policy makers and other interested stakeholders of the potential future risk posed by IFN-g test positive animals not going to slaughter, being left on the farm and being free to trade. The study presents important and unique results of the associated risk for bTB of IFN-g positive animals over time.

Future studies could include an exploration of farmer’s attitudes and motivations towards positive IFN-g animals and management of disease risks at the farm level. This could provide insights into the possible motivations of farmers, as part of disease control schemes where farmers are responsible for risk management decisions regarding infectious disease control.

As a conclusion, this study has shown that IFN-g positive animals that are not sent to slaughter and left on farms have a higher future risk of failing the tuberculin skin-test relative to IFN-g negative animals. Such animals are at increased risk of becoming bTB reactors and triggering breakdowns in herds in which they reside or to a new herd, if traded. These results highlight the need to review the current IFN-g scheme in Northern Ireland in terms of disclosing IFN-g positive animals that are not sent to slaughter. If neither enforcement nor restrictions apply to IFN-g positive animals detected on farms, these animals can be sold or moved freely and potentially spread bTB. We suggest as a minimum risk mitigation measure, that mandatory disclosure of the IFN-g test status of animals should be implemented, if animals are to be traded.

## Additional files

Additional file 1:
**Gamma negative animals.** A table is presented with the breakdown of Herd type and Sex by DVO region for Gamma negative animals. Additional file 2:
**Cox-proportional hazards model.** A Cox survival model of time to a subsequent positive skin-test for cattle within interferon-gamma tested problem cattle herds in Northern Ireland with a follow-up time of 18 months (550 days post-test; *n* = 10 517) is presented.
