# Supplementary material for: Should they stay, or should they go? Relative future risk of bovine tuberculosis for interferon-gamma test-positive cattle left on farms
Source: Vet Res. 2015 Sep 4;46(1):90. doi: 10.1186/s13567-015-0242-8 (PMC4559371; doi:10.1186/s13567-015-0242-8)
Supplement: Additional file 1: — Gamma negative animals. A table is presented with the breakdown of Herd type and Sex by DVO region for Gamma negative animals. [file 13567_2015_242_MOESM1_ESM.docx]

| **Region** | **Dairy** | | **Beef** | | **Total** |
| --- | --- | --- | --- | --- | --- |
|  | Male | Female | Male | Female |  |
| **N** | 193 | 3814 | 667 | 1513 | 6187 |
| **SW** | 175 | 3900 | 834 | 1837 | 6746 |
| **SE** | 352 | 5291 | 1084 | 2053 | 8780 |
| **Total** | 720 | 13005 | 2585 | 5403 | 21713 |
