# Supplementary material for: Should they stay, or should they go? Relative future risk of bovine tuberculosis for interferon-gamma test-positive cattle left on farms
Source: Vet Res. 2015 Sep 4;46(1):90. doi: 10.1186/s13567-015-0242-8 (PMC4559371; doi:10.1186/s13567-015-0242-8)
Supplement: Additional file 2: — Cox-proportional hazards model. A Cox survival model of time to a subsequent positive skin-test for cattle within interferon-gamma tested problem cattle herds in Northern Ireland with a follow-up time of 18 months (550 days post-test; n = 10 517) is presented. [file 13567_2015_242_MOESM2_ESM.docx]

| **Covariates** | **Hazard ratio** | ***P*-Value** | **95% Confidence Interval** | |
| --- | --- | --- | --- | --- |
| **Exposure**  **(*referent: Gamma negative*)** | |  | Lower | Upper |
| Gamma positive | 3.69 | <0.001 | 2.90 | 4.68 |
| **Herd type**  ***(referent: Dairy*)** | |  |  |  |
| Beef | 0.53 | <0.001 | 0.63 | 0.97 |
| **DVO region**  **(*referent: Southeast)*** | |  |  |  |
| North | 1.35 | 0.410 | 0.66 | 2.75 |
| Southwest | 1.99 | 0.056 | 0.98 | 4.02 |
| **Variance component** | |  |  |  |
| Theta | 3.75 | <0.001 |  |  |
